# C-type lectin-like domains in *Fugu rubripes*

**DOI:** 10.1186/1471-2164-5-51

**Published:** 2004-08-01

**Authors:** Alex N Zelensky, Jill E Gready

**Affiliations:** 1 Computational Proteomics and Therapy Design Group, John Curtin School of Medical Research, Australian National University, PO Box 334, Canberra, ACT 2601, Australia

## Abstract

**Background:**

Members of the C-type lectin domain (CTLD) superfamily are metazoan proteins functionally important in glycoprotein metabolism, mechanisms of multicellular integration and immunity. Three genome-level studies on human, *C. elegans *and *D. melanogaster *reported previously demonstrated almost complete divergence among invertebrate and mammalian families of CTLD-containing proteins (CTLDcps).

**Results:**

We have performed an analysis of CTLD family composition in *Fugu rubripes *using the draft genome sequence. The results show that all but two groups of CTLDcps identified in mammals are also found in fish, and that most of the groups have the same members as in mammals. We failed to detect representatives for CTLD groups V (NK cell receptors) and VII (lithostathine), while the DC-SIGN subgroup of group II is overrepresented in *Fugu*. Several new CTLD-containing genes, highly conserved between *Fugu *and human, were discovered using the *Fugu *genome sequence as a reference, including a CSPG family member and an SCP-domain-containing soluble protein. A distinct group of soluble dual-CTLD proteins has been identified, which may be the first reported CTLDcp group shared by invertebrates and vertebrates. We show that CTLDcp-encoding genes are selectively duplicated in *Fugu*, in a manner that suggests an ancient large-scale duplication event. We have verified 32 gene structures and predicted 63 new ones, and make our annotations available through a distributed annotation system (DAS) server  and their sequences as additional files with this paper.

**Conclusions:**

The vertebrate CTLDcp family was essentially formed early in vertebrate evolution and is completely different from the invertebrate families. Comparison of fish and mammalian genomes revealed three groups of CTLDcps and several new members of the known groups, which are highly conserved between fish and mammals, but were not identified in the study using only mammalian genomes. Despite limitations of the draft sequence, the *Fugu rubripes *genome is a powerful instrument for gene discovery and vertebrate evolutionary analysis. The composition of the CTLDcp superfamily in fish and mammals suggests that large-scale duplication events played an important role in the evolution of vertebrates.

## Background

The superfamily of proteins containing the C-type (Ca-dependent) lectin-like domain (CTLD) is a large group of extracellular proteins characterized by evolutionary flexibility and functional versatility [1,2]. Its members have been extensively studied because of their involvement in diverse physiological processes, and their ability to bind selectively a wide variety of ligands. As the superfamily name suggests, carbohydrates (in various contexts) are primary ligands for CTLDs and this binding is Ca-dependent [3]. However, the fold has been shown to specifically bind proteins [4], lipids [5] and inorganic compounds including CaCO_3 _and ice [6-9]. In several cases, the domain is multivalent and may bind both protein and sugar [10-12].

Three studies using the whole-genome approach have been published analyzing the distribution of the superfamily in *C. elegans *[13], *D. melanogaster *[14] and human [15]. An early study [2] attempted to generalize findings on vertebrate CTLD-containing proteins (CTLDcps), and to classify them into groups. This classification included 7 groups and, although not sufficient to describe later known CTLDcps even in mammals and other vertebrates, has been widely used by CTLD researchers. The recent work of Drickamer and Fadden [15] provided an updated classification of human and mouse CTLDcps, based on a comprehensive analysis of CTLDcps encoded by the human genome; this comprises 14 groups. These whole-genome studies and genome annotation projects demonstrated the relative abundance of CTLDcps and importance of the domain.

### Known fish C-type lectins

A number of fish CTLDcp sequences have been reported separately in the literature and public sequence databases. The best-studied and most distinct set are serum antifreeze proteins (AFPs) from several cold-water-living species [7,16,17]. These sequences consist mostly of just a CTLD, and were classified as group VII members based on domain architecture. A three-dimensional structure of the sea raven antifreeze protein has been determined experimentally [18].

Apart from AFPs, several other soluble bony-fish CTLDcps have been described: 5 isoforms of *Salmo salar *serum lectin (SSL) [19], three collectins from different Cyprinidae carp family species [20], skin mucus protein AJL-2 [21] and two C-type lectins (eCL-1 and eCL-2) from gills of Japanese eel [22], two lectins from rainbow trout liver [23], a carp lectin [24], goldfish lectin OL-1 (GI: 26000685, unpublished), and a liver lectin from *Gillichthys mirabilis *(long-jawed mudsucker), annotated as "mannose receptor C" [25].

Known membrane-bound CTLDcps from bony fishes include a polycystic kidney disease protein 1 (PKD1) orthologue from *Fugu *[26], a rainbow trout Kupffer cell receptor homologue [27], and a set of putative killer cell receptors (KLR) identified recently [28]. Although predicted coding sequences for CTLDcps from winter flounder (GI:28394504, unpublished) and medaka fish [29] do not contain a recognizable transmembrane (TM) domain, based on CTLD sequence and, in the case of the medaka CTLDcp, domain structure, they should be assigned to group II, as the absence of TM regions may be a result of incomplete prediction.

The only known CTLDcp sequence from cartilaginous fishes is a tetranectin homologue from reef shark cartilage [30].

### Fugu genome sequence

The *Fugu rubripes *genome, available since 2002 [31], is the second vertebrate genome sequenced. It is 8 times smaller than the human genome and is proving to be an effective instrument in analyzing the human genome because of its compactness, low content of repetitive elements and the relatively large evolutionary distance between fish and mammals, which is estimated to be about 430 Myr [32].

Currently three versions of the *Fugu rubripes *genome assembly are publicly available. The second version of the assembly (v.2), constructed from 4.1 million sequencing reads (5.4 X sequence coverage), was reported in the original publication announcing the completion of the *Fugu rubripes *genome sequencing [31]. The third version (v.3) was released in August 2002, has slightly better coverage (5.7X) and improved scaffold contiguity. Sequence data for all three assembly versions can be downloaded from the Joint Genome Institute web site [33]. The JGI site and the EnsEMBL web site [34] are the two main portals to the *Fugu rubripes *genome annotation. Although EnsEMBL and JGI annotations and genome browsers are different, they share the same gene and transcript structure predictions created by the EnsEMBL pipeline.

Several analyses of the draft *Fugu *genome sequence targeting different protein families have been published recently [35-39], which showed its usefulness for evolutionary and functional studies as well as gene discovery. Here we present an analysis of the presence of the CTLD superfamily in the draft assembly of the *Fugu rubripes *genome.

## Results

### Comparison of assembly versions 2 and 3

At the time this study was started, annotation of the v.3 assembly was not yet published; hence, most of our analysis was done with v.2 of the assembly and later mapped to the v.3 assembly. From our experience, there is no substantial difference between v.2 and v.3 assemblies in the amount of sequence information and its quality, although the v.3 assembly contains longer scaffolds due to more extensive linkage. Despite very high similarity at the sequence level, the v.3 assembly annotation contains no history information that would provide links between contigs, genes, and transcripts in the second and the third versions of the assembly. None of the stable identifiers for genes, transcripts or peptides from v.2 are present in v.3. This information cannot be generated by usual procedures used in EnsEMBL (e.g. ID Mapping Application, which is a part of EnsEMBL Java APIs) and has to be obtained by sequence comparisons. This lack of correspondence creates difficulties for the sequence analyzer and end point reader. To facilitate analysis and allow comparison, references to feature identifiers for both of the assemblies are given in Table 1.

**Table 1:** CTLD-encoding genes identified in the Fugu rubripes genome.^a^

**Name**	**Description**	**v.2 gene ID**	**v.3 gene ID**
**I Hyalectans**			

AGGRECAN	AGGRECAN	ANUFRUG00000000095	ANUFR2G00000000089
AGGRECAN-F1	Fugu aggrecan paralogue	ANUFRUG00000000081	ANUFR2G00000000077
BREVICAN	BREVICAN	SINFRUG00000078610	SINFRUG00000151617
BREVICAN-F1	Fugu brevican paralogue	SINFRUG00000074933	SINFRUG00000128229, SINFRUG00000128230, SINFRUG00000128231
NEUROCAN	NEUROCAN	SINFRUG00000054833	SINFRUG00000150572, SINFRUG00000150573, SINFRUG00000150574, SINFRUG00000150576
NEUROCAN-F1	Fugu Neurocan paralogue	ANUFRUG00000000142	ANUFR2G00000000154
VERSICAN	VERSICAN	ANUFRUG00000000144	ANUFR2G00000000164
VERSICAN-F1	Fugu versican paralogue (fragment containing EGF, CTLD and CCP domains)	ANUFRUG00000000061	ANUFR2G00000000059
VERSICAN-F2	Fugu versican paralogue (fragment containing link and Ig domains)	ANUFRUG00000000043	ANUFR2G00000000041

**II Dendritic cell receptors, mono-ctld macrophage receptors, ASGR**			

DC-SIGN-F1	Fugu DC-SIGN paralogue	ANUFRUG00000000029	ANUFR2G00000000027
DC-SIGN-F2	Fugu DC-SIGN paralogue	ANUFRUG00000000067	ANUFR2G00000000063
DC-SIGN-F3	Fugu DC-SIGN paralogue	ANUFRUG00000000069	ANUFR2G00000000065
DC-SIGN-F4	Fugu DC-SIGN paralogue	ANUFRUG00000000071	ANUFR2G00000000067
DC-SIGN-F5	Fugu DC-SIGN paralogue	ANUFRUG00000000073	ANUFR2G00000000069
DC-SIGN-F6	Fugu DC-SIGN paralogue	ANUFRUG00000000109	ANUFR2G00000000105
DC-SIGN-F7	Fugu DC-SIGN paralogue	ANUFRUG00000000085	ANUFR2G00000000123
DC-SIGN-F8	Fugu DC-SIGN paralogue	ANUFRUG00000000087	ANUFR2G00000000081
DC-SIGNR	DCSIGN receptor	ANUFRUG00000000027	ANUFR2G00000000025
HML2	Similar to human macrophage lectin	SINFRUG00000060881	SINFRUG00000120587
SRCL	Scavenger receptor with C-type lectin	SINFRUG00000071148	SINFRUG00000134389
SRCL-F1	Putative Fugu paralogue of SRCL	SINFRUG00000064389	SINFRUG00000152316
XLCMCL	eXtra Large Coiled coil region containing Membrane C-type Lectin	ANUFRUG00000000053	ANUFR2G00000000051

**III Collectins**

COLEC10	COLEC10	SINFRUG00000077039	SINFRUG00000125405
**MGC3279**	**Uncharacterized collectin family member**	SINFRUG00000064196	SINFRUG00000147955

**IV Selectins**			

SELECTIN-E	E-Selectin	ANUFRUG00000000001	ANUFR2G00000000001
SELECTIN-L	L-SELECTIN	ANUFRUG00000000003	ANUFR2G00000000003
SELECTIN-P	P-SELECTIN	ANUFRUG00000000005	ANUFR2G00000000005

**VI Multi-CTLD molecules. Macrophage Mannose Receptor (MMR) family**			

DEC205	DEC205	ANUFRUG00000000011	ANUFR2G00000000011
Endo180	Endo180	SINFRUG00000058766	SINFRUG00000152106
MManR	Macrophage mannose receptor	SINFRUG00000071196	SINFRUG00000126868, SINFRUG00000134363
MManR-F1	Fugu mannose receptor paralogue (fragment)	SINFRUG00000064600	SINFRUG00000152797
MManR-F2	Fugu macrophage mannose receptor paralogue.	ANUFRUG00000000039	ANUFR2G00000000035 ANUFR2G00000000037
MManR-F3	Fugu paralogue of MMR-family gene	SINFRUG00000066378	SINFRUG00000152288
MManR-F4	Fugu paralogue of MMR-family gene (fragment)	SINFRUG00000078047	SINFRUG00000152861
MManR-F5	Fugu MMR-family member (fragment)	ANUFRUG00000000091	ANUFR2G00000000085
PLA2R	Phosopholipase A2 receptor	ANUFRUG00000000009	ANUFR2G00000000009

**VIII MT-75, layilin**			
LAYILIN	Layilin	ANUFRUG00000000089	ANUFR2G00000000083
LAYILIN-F1	Fugu layilin paralogue	ANUFRUG00000000075	ANUFR2G00000000071
MT-75	MT-75	SINFRUG00000084745	SINFRUG00000145404

**IX Tetranectin family**			

CLECSF1	CLECSF1	SINFRUG00000050048	SINFRUG00000136890
SCGF	SCGF	ANUFRUG00000000125	ANUFR2G00000000121
TETRANECTIN	Tetranectin	SINFRUG00000084961	SINFRUG00000144710
TETRANECTIN-F1	Fugu tetranectin paralogue	SINFRUG00000083037	SINFRUG00000149544

**X PKD**			

PKD1	Polycystic kidney disease protein 1	SINFRUG00000033997	
PKD1L2	PKD-1 homologue 2	ANUFRUG00000000121	ANUFR2G00000000117

**XI Attractin family**			

ATTRACTIN	Attractin	SINFRUG00000071911	SINFRUG00000136030
ATTRACTIN-F1	Fugu paralogue of Attractin	SINFRUG00000060472	SINFRUG00000147061
**KIAA0534**	**KIAA0534**	SINFRUG00000056251	SINFRUG00000121439

**XII Eosinophil major basic protein family**			

EMBPL	Putative Fugu EMBP-like protein	ANUFRUG00000000023	ANUFR2G00000000021

**XIII DGCR family**			

DGCR2	DGCR2	SINFRUG00000082125	SINFRUG00000155593

**XIV Thrombomodulin family**			

C1qRP	C1qRP	ANUFRUG00000000049	ANUFR2G00000000047
C1qRP-F1	Putative Fugu C1qRP paralogue (fragment)	ANUFRUG00000000013	disappeared
**CETM**	**Protein containing CTLD, EGF and transmembrane domains**	ANUFRUG00000000057	ANUFR2G00000000055
ENDOSIALIN	ENDOSIALIN	ANUFRUG00000000117	ANUFR2G00000000113
THROMBOMOD	Thrombomodulin	SINFRUG00000077807	SINFRUG00000153798

**XV Bimlec**			

**BIMLEC**	**Novel C-type lectin from BCG cell wall induced monocyte**	ANUFRUG00000000007	ANUFR2G00000000007

**XVI SEEC**			

**SEEC**	**Novel SCP-EGF-EFG-CTLD containing protein.**	ANUFRUG00000000041	ANUFR2G00000000039

**XVII CBCP**			

**CBCP**	**Calx-Beta and CTLD containing protein**	ANUFRUG00000000047	ANUFR2G00000000045

**AFP Antifreeze protein**			

AFPL-F1	Antifreeze protein-like	ANUFRUG00000000045	ANUFR2G00000000043
AFPL-F2	Antifreeze protein-like	ANUFRUG00000000139	disappeared

**F1 Fugu dual-CTLD molecules**			

FDC-F1	Putative Fugu dual-CTLD protein 1	ANUFRUG00000000025	ANUFR2G00000000023
FDC-F2	Putative Fugu dual-CTLD protein 2	ANUFRUG00000000037	ANUFR2G00000000033
FDC-F3	Putative Fugu dual-CTLD protein 3	ANUFRUG00000000099	ANUFR2G00000000093
FDC-F4	Putative Fugu dual-CTLD protein 4	ANUFRUG00000000103	ANUFR2G00000000097, ANUFR2G00000000099
FDC-F5	Putative Fugu dual-CTLD protein 5	ANUFRUG00000000107	ANUFR2G00000000103
FDC-F6	Putative Fugu dual-CTLD protein 6	ANUFRUG00000000123	ANUFR2G00000000119
FDC-F7	Putative Fugu dual-CTLD protein 7	ANUFRUG00000000101	ANUFR2G00000000095
FTCP	Putative Fugu triple-CTLD protein	ANUFRUG00000000015	ANUFR2G00000000013

**L Link domain**			

BRAL1	Brain link protein-1	SINFRUG00000078615	SINFRUG00000151615
CD44	CD44	ANUFRUG00000000113	ANUFR2G00000000109
CRTL1	Cartilage linking protein 1	SINFRUG00000078961	SINFRUG00000137046
CRTL1-F1	Putative fugu cartilage linking protein paralogue	ANUFRUG00000000059	ANUFR2G00000000057
CRTL1-F2	Putative fugu cartilage linking protein paralogue	SINFRUG00000074643	SINFRUG00000142167, SINFRUG00000142169, SINFRUG00000142171
HAPLN3	Hyaluronan and proteoglycan link protein 3	SINFRUG00000052853	SINFRUG00000155413
HAPLN3-F1	Putative Fugu paralogue of HAPLN3	SINFRUG00000079552	SINFRUG00000129575
Lyve-1	Lymphatic vessel endothelial HA receptor-1	ANUFRUG00000000077	ANUFR2G00000000073
STABILIN-1	Stabilin-1	ANUFRUG00000000079	ANUFR2G00000000075
STABILIN-2	Stabilin-2	SINFRUG00000074867	SINFRUG00000146665
TSG-6	TSG-6	SINFRUG00000075173	SINFRUG00000148136

**NLSLH**			

**NLSLH**	**Novel L-SeLectin Homologue**	ANUFRUG00000000055	ANUFR2G00000000053
NLSLH-F1	Fugu CTLD containing gene fragment, NLSLH paralogue	ANUFRUG00000000097	ANUFR2G00000000091

**U Unclassified**			

AGGRECOL	Putative Fugu CTLD-containing protein equally similar to aggrecan and placenta collectin.	ANUFRUG00000000083	ANUFR2G00000000079
ANZG001	Putative Fugu CTLD-containing protein (fragment)	ANUFRUG00000000019	ANUFR2G00000000017
ANZG002	Putative Fugu CTLD-containing protein (fragment)	ANUFRUG00000000021	ANUFR2G00000000019
ANZG004	Putative Fugu protein with CTLD and FTP domains	ANUFRUG00000000093	ANUFR2G00000000087
ANZG005	Putative Fugu CTLD-containing protein (fragment)	ANUFRUG00000000065	disappeared
ANZG006	Putative Fugu CTLD-containing protein (fragment)	ANUFRUG00000000111	ANUFR2G00000000107
ANZG007	Putative Fugu CTLD-containing protein (fragment)	ANUFRUG00000000063	ANUFR2G00000000061
ANZG008	Putative Fugu CTLD-containing protein (fragment)	ANUFRUG00000000017	ANUFR2G00000000015
ANZG010	Putative Fugu CTLD-containing protein	ANUFRUG00000000051	ANUFR2G00000000049
ANZG011	Putative Fugu CTLD-containing protein	ANUFRUG00000000115	ANUFR2G00000000111
CFN3	Protein with CTLD and FN3 domains.	ANUFRUG00000000105	ANUFR2G00000000101
DEC205-FUSE	Large Fugu protein which looks like a DEC205 fused to another CTLD-containing gene	ANUFRUG00000000119	ANUFR2G00000000115
FG75645	Fugu CTLD-containing protein fragment	SINFRUG00000075645	SINFRUG00000139863
PTP-GMC1	Protein-tyrosine phosphatase expressed by glomerular mesangial cells	ANUFRUG00000000130	ANUFR2G00000000137

^a ^All *Fugu *CTLDcps identified in this analysis are listed. Columns 3 and 4 contain stable identifiers for gene models in the v.2 and v.3 assembly databases, respectively. Identifiers starting with ANUFRU and ANUFR2 belong to our predictions on the v.2 and v.3 assemblies, respectively, and are underlined. EnsEMBL gene stable identifiers are given if the original predictions were used. Bolded members denote *Fugu *proteins matched with novel human orthologues.

### Protein database searches

Due to almost complete lack of cDNA or EST sequences for *Fugu rubripes*, most of the EnsEMBL gene structure predictions are based on homology with known protein sequences from other organisms, mostly mammals. We expected a significant fraction of CTLDcps to be conserved between fish and human, and, therefore, to be predicted correctly by EnsEMBL in the *Fugu *genome. So our first approach to detecting *Fugu *CTLDcps was to search a sequence database of predicted *Fugu *proteins with a hidden Markov model (HMM) for the CTLD. This search returned 69 significant matches. Some of the identified genes had a description assigned to them, apparently derived from the description of the sequence they were found to be homologous to. These descriptions, however, could not be used as a reliable basis for assigning orthology and paralogy relationships. For example, a sequence, which we later identified as an Endo180 orthologue (SINFRUG00000058766 in v.2 assembly annotation) is described as "80 KDA SECRETORY PHOSPHOLIPASE A2 RECEPTOR PRECURSOR PLA2", while another gene, which we designated as an aggrecan orthologue (SINFRUG00000069597 in v.2 annotation) was annotated as "ADRENOLEUKODYSTROPHY PROTEIN (ALDP)". Therefore, we reviewed domain architecture and sequence similarity matches for each of the sequences found to verify phylogenetic relationships.

### Homology detection

The results of Inparanoid [40] comparison (see Methods) of all human to all *Fugu *CTLDcps were used to initially cluster the set of *Fugu *proteins and detect approximate orthology/paralogy links. Inparanoid has an important advantage over phylogenetic tree reconstruction software, as it does not require a multiple alignment of sequences but creates a distance matrix of the local pairwise alignments. This method assigned putative human orthologues to 25 *Fugu *proteins. Orthology relationships for the other 44 sequences from the set were established by individual analyses.

### Revision of CTLDcp gene structure predictions

While analyzing phylogenetic relationships predicted by Inparanoid, we discovered several systematic and sporadic mistakes in the EnsEMBL gene predictions. The most widespread mistake was a failure to include exons encoding TM domains into gene structure prediction. Consequently, almost all EnsEMBL-predicted *Fugu *CTLDcps were soluble proteins, whereas very few human CTLDcps are. Simple comparison with the GenScan [41] features overlapping the CTLD-encoding genes showed that absence of TM domains is a result of coding sequence (CDS) mis-prediction rather than a fundamental difference in *Fugu *CTLDcps. GenScan predictions, in turn, could not be used as a basis for our analysis because they sometimes contain regions that are absent from human or mouse orthologues, and often merge neighboring genes. Another general problem was observed with proteins that had a previously unknown domain architecture (see below). In such cases individual domains were split into separate gene models.

**Figure 1:**
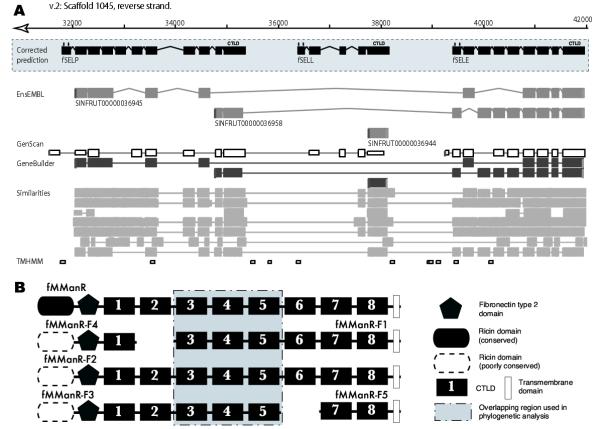
**Fugu genome sequence and annotation. ***A. Fugu *selectin gene cluster annotation in the EnsEMBL database (v.2 annotation is shown, v.3 annotation is almost identical to v.2). Gene models predicted by us based on comparison with human selectins are shown in the grey box. As shown, the CTLD is encoded by the 5' exon in fSELP, fSELL and fSELE; the TM segment is encoded by the 3' exon. EnsEMBL predicted transcripts, GenScan predictions and similarity features are shown on the tracks below. Stable IDs for EnsEMBL transcripts are given. The TMHMM track shows ORFs encoding TransMembrane regions predicted by the TMHMM program (see Methods). *B. *Fragments of group VI genes found on various scaffolds. CTLD numbers indicate sequential number of CTLD in full-length MManR, while numbers for the CTLD in the partial sequences indicate the MManR CTLD sequence they are most closely homologous to.

In addition to these systematic problems, there were multiple sporadic ones. For example, our analysis of the *Fugu *genome shows that, similarly to the human and mouse genomes, the selectin cluster is well conserved and contains all three selectin genes in tandem (SELE, SELL, SELP), located on scaffolds 1045 (32046–41921) and 166 (83937–93826) in the v.2 and v.3 *Fugu *genome annotations, respectively. However, the EnsEMBL annotation contains a prediction of two overlapping genes (v.2: SINFRUG00000085188 and SINFRUG00000085187; v.3: SINFRUG00000123102 and SINFRUG00000123101), one of which is located in the intron of the other (Figure 1).

To solve these problems, we had to manually revise the predicted structure for all genes encoding proteins detected by the protein-level searches, and correct them using supporting evidence available in the EnsEMBL database, as well as additional evidence generated by us. The latter included similarity features produced by genome-wide GeneWise and BLAST searches with CTLD profiles and sequences, transmembrane domain predictions, and similarity matches to the complete sequence of supposed human or mouse orthologues.

As the final stage of the CTLDcp identification process, we performed a set of DNA-level comparisons to ensure that the CTLD-containing loci that are not covered by EnsEMBL-predicted genes, or for which transcript predictions are wrong and, thus, not detectable by protein database searches, were not omitted from the analysis. This "quality control" step led to identification of an additional set of 25 well conserved CTLDcps, which had both new and known domain architectures, as well as additional individual CTLDs, which were merged with neighboring CTLDcp loci if appropriate.

### Groups of *Fugu *CTLDcps

After all these searches, we had identified a set of 94 *Fugu rubripes *loci encoding CTLDcps (Table 1), which in total contain 173 individual CTLDs, including PTR/Link-type CTLDs [42]. *Fugu *CTLDcps were named according to their human orthologues, established on the basis of domain composition and sequence similarities. Where more than one homologue was present in *Fugu*, a name was produced by adding a suffix of the form "-FXX", where XX is a sequential number of the paralogue, to the name of the closest human homologue. Predicted CTLDcps that do not have homologues among the known CTLDcps have identifiers of the form ANZ000. A few of these novel genes were orthologous to loci in other vertebrate genomes supported by expression data, but otherwise are un-characterized, and were assigned descriptive names (CBCP, Bimlec, SEEC, CETM, NLSLH).

**Figure 2:**
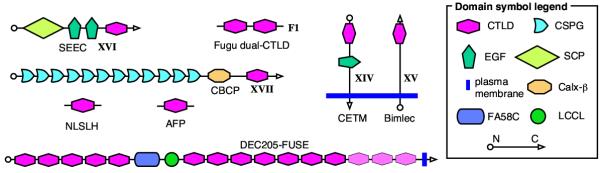
**CTLDcps with novel domain architectures. ***Fugu *CTLD-containing proteins, which do not fit into the existing CTLDcp classification are shown. Domain abbreviations are explained in the text. Roman numbers near names indicate suggested new group names for the new *Fugu *sequences, which also have new predicted human homologues. C-terminal CTLDs of DEC205-FUSE that are not present in the v.3 assembly are shown in light pink.

We have clustered *Fugu *CTLDcps using the classification scheme for human CTLDcps based on domain composition; this comprises 14 groups [15]. Link/PTR-domain-containing CTLDcps, apart from hyalectans, were placed into a separate group. Among the *Fugu *CTLDcps that did not have mammalian homologues we detected a distinct group of soluble dual-CTLD sequences, which we have called F1 (Figure 2). The remainder of the *Fugu*-specific CTLDcps were assigned to the U (Unclassified) group. Gene structure prediction for members of the U group is the lowest in quality, due to lack of supporting evidence apart from similarity to CTLD sequence profiles and GenScan predictions.

**Figure 3:**
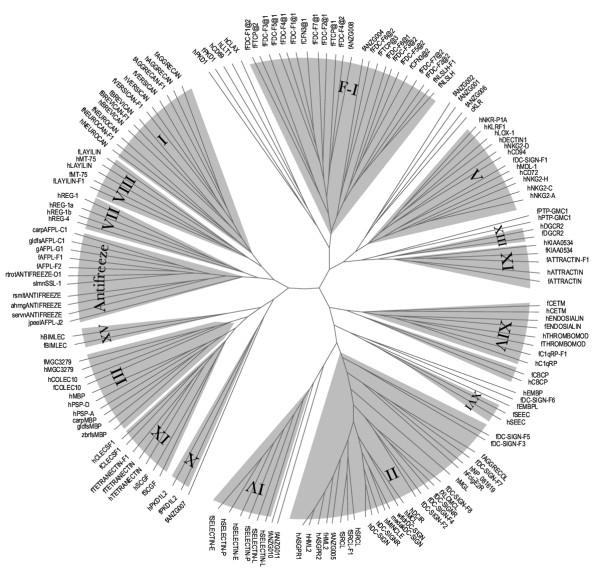
**Phylogenetic relationships between fish and human CTLDs. **A phylogenetic tree built on a ClustalW alignment of a 95% non-redundant collection of predicted *Fugu *CTLDs and known human and fish CTLDs. Link domains and group VI CTLDs were excluded from the alignment. The tree was built by the neighbor-joining method with 100 bootstrap trials using the ClustalW program. PhyloDraw was used to draw the radial cladogram shown. Branches containing CTLDs from CTLDcps belonging to the same group are shaded; group numbers are marked. Lower case prefixes in the identifiers indicate taxonomic origin: h – *Homo sapiens*, f – *Fugu rubripes*, zbrfs – *Danio rerio *(zebrafish), g – *Gillichthys mirabilis*, gldhs – *Carassius auratus *(goldfish), carp – *Cyprinus carpio *(common carp), rsmlt – *Osmerus mordax *(rainbow smelt), slmn – *Salmo salar *(Atlantic salmon), wfldr – *Pseudopleuronectes americanus *(winter flounder), ahrng – *Clupea harengus *(Atlantic herring), servn – *Hemitripterus americanus *(sea raven), jpeel – *Anguilla japonica *(Japanese eel), medak – *Oryzias latipes *(Japanese medaka), c – *Paralabidochromis chilotes *(cichlid fish).

All but two groups of human CTLDcps have detectable representatives in the *Fugu *genome (Figure 3, Table 1). We did not detect any orthologues for groups V (NK cell receptors) and VII (lithostathine/Reg family). The member repertoire for most of the other groups is very well conserved between *Fugu *and human. However, groups II and III, which include some of the best-studied mammalian CTLDcps, have a significantly different member composition in *Fugu*. In summary:

#### Group I

All four members of the lectican group that are present in human have orthologues in the *Fugu *genome. Each of the *Fugu *hyalectan genes is duplicated. One of the *Fugu *versican copies is split between two scaffolds in the v.2 assembly.

#### Group II

We found only one representative of the asialoglycoprotein receptor (ASGR) family in *Fugu *(HML2), while in human this family has 3 members encoded by a gene cluster on Ch 17 (ASGR1, ASGR2, HML2). The *Fugu *sequence was identified as an HML2 orthologue by phylogenetic analysis based on the alignment of CTLD sequences. Another clearly identifiable member of group II is the orthologue of scavenger cell receptor C-type lectin (SRCL), which is duplicated in *Fugu *and is 50% identical to the human SRCL. The rest of the group II *Fugu *CTLDcps (DC-SIGN-F1 – DC-SIGN-F8, XLCMCL) do not have clearly identifiable orthologues among known human CTLDcps, although phylogenetic analyses based on CTLD sequence alignment indicate that they are homologous to members of the group II subgroup containing DC-SIGN, Mincle and Dectin-2, which also appear as top hits in BLAST searches. However, this subset of group II *Fugu *sequences co-clusters in phylogenetic trees and is not similar enough to any tetrapod sequence to establish orthology. Four of the sequences (DC-SIGN-F2, DC-SIGN-F3, DC-SIGN-F4, DC-SIGN-F5) are located in a cluster on scaffold 75 in the v.3 assembly. Two members of the subgroup (DC-SIGN-F1 and DC-SIGN-F6) have unstable placements in phylogenetic trees, and may appear on a branch containing human/mouse group V sequences, if the latter are included in the alignment. This association is, however, unstable and may be due to mistakes in CDS prediction or phylogeny reconstruction. Alternatively, it is possible that these sequences are homologous to the common predecessor of group V and group II CTLDcps.

#### Group III

Although *Fugu *has two collectins, there are no orthologues for mannose binding proteins (MBPs) or pulmonary surfactant proteins (PSP), which are the best studied members of the group in human. Both of the *Fugu *collectins (COLEC10, MGC3279) are well conserved compared with their human orthologues and co-cluster with them in phylogenetic trees. No functional information is available for the novel collectin MGC3279, which was discovered in a large-scale cDNA sequencing project and maps to chromosome 2p25.3 in the v.31 NCBI assembly of the human genome, but the exceptionally high level of conservation between human and fish (~76% identity) strongly suggests that it is functional and important in both organisms. COLEC10 (collectin liver 1, CL-L1) was originally reported as limited to birds and mammals [43] based on the Zoo-blot analysis.

#### Group IV

As already mentioned, all three selectin genes found in other vertebrates are present in *Fugu *and have the same genome arrangement.

#### Group VI

We identified *Fugu *orthologues for all four human group VI members: macrophage mannose receptor (MManR), DEC-205 (CD205), phospholipase A2 receptor (PLA2R) and Endo180. In addition, there are 5 sequences (MManR-F1 – MManR-F5) showing high similarity to members of the group, four of which do not contain the minimal number of CTLDs (8) present in the known group VI sequences (Figure 1). The fragments belong to at least 3 group VI CTLDcps. Although the most parsimonious explanation of the presence of these fragments would be that each of the genes encoding an eight-CTLD molecule (MManR, Endo180 and PLA2R) was copied in a chromosome or genome duplication event, phylogenetic analysis indicates that all five sequences are paralogues of the MManR gene, which, thus, appears to have been duplicated several times.

There is one more potential group VI member in *Fugu*. A GenScan-predicted DEC-205-FUSE gene, which was assigned to the U group, encodes a large protein (~2000 residues) with multiple CTLDs clustered in two groups: 5 at the N terminus and 10 (7 in v.3 assembly) at the C terminus, with an LCCL domain [named after its presence in Limulus factor C, cochlear protein Coch-5b2, and late gestation lung protein Lgl1; [44]] and a coagulation factor 5/8 C-terminal domain (discoidin domain, FA58C) lying in the middle separating the two groups of CTLDs (see Figure 2). EnsEMBL predictions in the DEC-205-FUSE locus in both versions of the assembly contain a large (4 kb) intron in the region encoding LCCL, FA58C and 8 CTLDs at the center of the molecule. LCCL has been observed in a combination with a CTLD in an invertebrate protein [45], while FA58C has been found only in combination with LCCL, but not with a CTLD [46]. Although there is no supporting cDNA or EST evidence for our predicted gene structure, the small intron sizes (e.g. LCCL is separated by 135 bp from the downstream CTLD) and well-conserved CTLDs, suggest that the prediction may be correct if the corresponding region was correctly assembled. There is no orthologue for DEC-205-FUSE in the human genome.

#### Groups VIII and IX

We have identified *Fugu *orthologues for all known human members of groups VIII and IX. One member in each of these groups is duplicated in *Fugu *(Layilin and Tetranectin).

#### Group X

In addition to the PDK1 orthologue, which was identified previously [26], there is at least one more putative group X member, orthologous to a recently identified human and mouse PKD1 homologue PKD1L2 [47]. It is interesting to note that the GenScan-predicted *Fugu *PKD1L2 sequence is very similar to the sequences of human and mouse PKD1L2 cDNAs, even though the latter were deposited in GenBank at the beginning of June 2003 – after GenScan prediction. This example indicates that *ab initio *GenScan predictions on the *Fugu *genome can be very accurate.

#### Group XII

We found a single sequence resembling mammalian eosinophil major basic proteins (EMBPs) in *Fugu *(EMBPL). Although the similarity between the mammalian and the fish sequences is very low (~30% identity), several observations suggest that the *Fugu *EMBP-like sequence is an orthologue of one of the two mammalian genes. First, the overall domain architecture of the fish protein is similar to that of the EMBPs. Although the fish CTLD has a neutral pI (7.1), it is preceded by a 30-residue peptide with a predicted pI of 3.62, analogous to the longer acidic neck of the mammalian EMBPs. In the existing classification [15], the presence of the acidic neck is used as the defining feature of group XII distinguishing it from the other group of single-CTLD soluble proteins (VII). Second, in the phylogenetic trees EMBPL usually appears on the same branch as EMBPs (e.g. Figure 3), albeit with low bootstrap support. Third, the exon-intron structure of the CTLD region is identical in fish and mammalian genes. Finally, the fish sequence has the same rare substitution in the fourth position of the WIGL motif as the EMBP sequences (discussed in more detail below).

#### Group XIV

The thrombomodulin family is fully represented in *Fugu*, with one gene duplicated (C1qRP). In addition, a novel member of the family conserved between *Fugu *and mammals was identified, which we named CETM (for CTLD, EGF, TransMembrane domain) (see Figure 2). Multiple full-length cDNA and EST sequences from different tissues found in nucleotide databases indicate that mammalian CETM is ubiquitously expressed. The sequence of the CETM CTLD contains a putative carbohydrate-binding motif (EPN), which is normally associated with mannose specificity.

#### Antifreeze-protein-like sequences

We identified two putative CTLDcp-encoding loci with similarity to antifreeze proteins: AFPL-F1 and AFPL-F2 (antifreeze-protein-like), almost identical to each other and positioned in tandem on scaffold 1930 in the v.2 assembly. In v.3 of the assembly, the AFPL-encoding region was rearranged and one of the AFPL loci disappeared. The intron-exon structure of the CTLD-encoding region is identical to the structure of the sea raven antifreeze protein gene [48] with three intron insertions (upstream of C1, downstream of the WIGL motif, and between C2 and C3 [49]), and very similar to the structure of the *Salmo salar *serum lectins [19], where only the first two splice sites are present. The Fugu AFPL gene expression is confirmed by an EST sequence BU806418, which covers the whole predicted CDS.

#### Link domain containing CTLDcps

All link domain-containing proteins identified in mammals are represented in *Fugu *and often are highly conserved between fish and human (e.g. TSG-6, 72%; Stabilin-1, 45% identity); we will consider them as a single group despite their different domain architectures. Predicted members of the CD44 family (CD44 and lymphatic vessel endothelium-specific hyaluronan receptor (Lyve-1)), however, are much more divergent from their human homologues, and it is not clear whether the two loci found in *Fugu *are orthologues of the two human genes or paralogues which arose by duplication of an ancestral gene.

In a recently published comprehensive study of another family of the Link group, the hyaluronan and proteoglycan binding link proteins (HAPLN), four homologues were identified in vertebrates (mouse, human and partially zebrafish) each linked to one of the four lecticans [50]. As all lecticans (i.e. group I) are duplicated in *Fugu*, we were expecting to also find duplicate copies of all HAPLN members. However, orthologues of only three HAPLNs were found (CRTL1, BRAL1, HAPLN3), two of which are linked to hyalectans in the same way as in mammalian genomes (CRTL1 with Versican, BRAL1 with Brevican). The state of the assemblies does not allow to determine conclusively whether HAPLN3 is linked to Aggrecan or not. Only two of the *Fugu *lectican gene duplications are accompanied by corresponding HAPLN genes: Aggrecan-F1 is linked to HAPLN3-F1 and the CRTL1 paralogue is present downstream to Versican-F1 in two tandem copies (CRTL1-F1 and CRTL1-F2). In neither version of the assembly could the HAPLN4 homologue be identified downstream to Neurocan or Neurocan-F1. Sequence conservation levels within the HAPLN proteins compared with their human orthologues is quite high (e.g. 76% identity for CRTL1).

#### Fugu dual-CTLD CTLDcps

The members of this group are soluble proteins with two or three CTLDs, which we initially characterized as fragments of putative macrophage mannose receptor paralogues. However, phylogenetic analysis showed that these proteins constitute a separate group, with no mammalian orthologues detectable in sequenced genome and protein databases. The domain structure prediction is confirmed by three zebrafish cDNAs (CAE17649, CAE17650, CAE17651), which have the same domain organization, although conservation between zebrafish and *Fugu *sequences is only moderate (~30%). Another homologue with the same domain structure and similarity to the F1 group members, which was returned as the top-scoring hit by BLAST searches in the nrdb, is the SCARF2 protein from a planarian *Girardia tigrina *[51]. A hypothetical dual-CTLD protein from *Drosophila *(NP_609962), which presumably corresponds to the single member of group B in the *Drosophila *CTLDcp classification of Dodd and Drickamer [14], was also detected as a F1 homologue by BLAST.

### Novel CTLDcps conserved between *Fugu *and mammals

Discovering novel superfamily members in existing database sequences is one of the most important and exciting outcomes of a systematic computer-based study such as this. We predicted putative *Fugu *orthologues for several uncharacterized mammalian CTLDcps (Bimlec, MGC3279, KIAA0534, CETM, SEEC, CBCP, NLSLH) that are well conserved between *Fugu *and mammals. Most of the predictions were supported by mammalian cDNA sequences from public databases, but for two of them (NLSLH and CBCP) no full-length cDNA from any organism was found in DBs. The high level of genomic sequence conservation over evolutionary time from fish to human, as in the case of NLSLH, and the presence of partial cDNA and EST sequences from rodents and human, as in the case of CBCP, were strongly suggestive that the predictions are correct. The novel CTLDcps that could be attributed to one of the 14 known groups have been discussed in the preceding sections for the corresponding groups; those that do not fit into the existing classification are described below.

A large (~2100 aa) proteoglycan (CBCP), containing a set of chondroitin sulphate proteoglycan (CSPG) repeats [52], which are homologous to the NG2 ectodomain [53], a calcium-binding Calx-β domain [54] and a CTLD, is a novel member of a protein family which had not been reported previously to have members containing CTLDs; examples of this family also include the human MCSP/CSPG4 [55] and mouse FRAS1 [56] genes. The prediction was supported by three overlapping but incomplete cDNA sequences from human and mouse, high levels of conservation between human and *Fugu *(~50% identity), and the compact structure of the predicted *Fugu *gene. CBCP has been placed in a new CTLD group, XVII; its domain structure is shown in Figure 2. We have cloned a full-length cDNA of mouse CBCP confirming the domain structure predicted in this study (A.N. Zelensky, in preparation). The CTLD of CBCP lacks Ca-binding residues, and its long loop region is short, resembling that of the group V CTLDs.

Another protein with a novel domain organization, whose prediction is strongly supported by available cDNAs, is SEEC (SCP, EGF, EGF, CTLD-containing protein) (see Figure 2), which is well conserved between human and *Fugu*. Although not described in a publication, a full-length human SEEC cDNA (AK074773) was sequenced in the NEDO high-throughput sequencing project. The predicted *Fugu *SEEC is 63% identical to the human sequence. The sperm-coating glycoprotein (SCP) domain, which is present in a broad set of organisms from yeast and plants to mammals, but whose function is unknown [57], is rarely observed in combination with other domains in proteins; in only one other known protein (from sea urchin) is it found together with an EGF domain [58], and SEEC is the first example of a CTLD-SCP combination. The potential Ca/carbohydrate-binding motif (QPD) characteristic of galactose specificity is present in the CTLD. SEEC has been placed in a new CTLD group XVI.

A predicted protein named "novel L-selectin homologue" (NLSLH) because its CTLD is most similar to selectin CTLDs is duplicated in *Fugu *(NLSLH and NLSLH-F1) but only moderately conserved (32% identity) between *Fugu *and human. The putative human orthologue is located on Ch1q25.1 about 18 Mb further from the centromere than the selectin cluster and is supported only by EST sequences (AA912157, AA889574), but not cDNAs. No conserved domains except for the CTLD could be detected in the human and *Fugu *NLSLH loci so, if the predictions are correct, NLSLH is a soluble single-CTLD-containing protein. Carbohydrate-binding motifs are not present in the NLSLH and NLSLH-F1 CTLDs.

Finally, a type I transmembrane protein Bimlec, whose prediction is supported by a full-length human cDNA, was placed in a new group XV.

### Dating the CTLDcp duplications

We found 12 groups of unlinked *Fugu*-specific CTLDcp paralogues (Table 1), and attempted to determine the duplication dates using two approaches: (1) based on the estimation of the number of synonymous nucleotide substitutions (Ks) in the coding sequences and (2) based on the molecular clock hypothesis.

**Figure 4:**
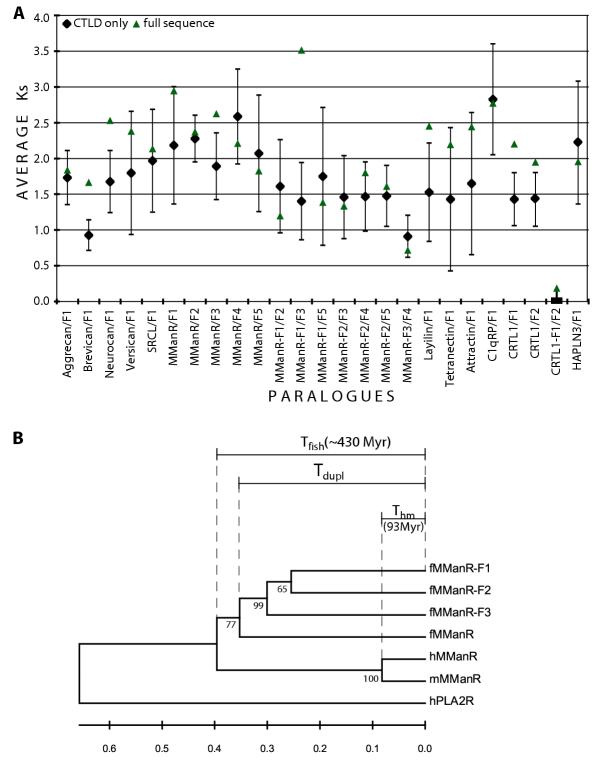
**CTLDcp duplication dates. **A. Average number of synonymous substitutions per synonymous site (Ks) for CTLDcp paralogue pairs based on full-sequence (triangle) and CTLD-only (diamond) alignments, measured with four different methods (see Methods). Error bars show one standard deviation in the CTLD-only measurements. All possible pairwise alignments between the MManR fragments and between the three CRTL1 paralogues were analyzed. Only homologous regions were used for MManR fragment alignments. B. A linearized phylogenetic tree built by the neighbor-joining method from Poisson-corrected distances between ClustalW-aligned sequences of CTLDs 3–5 from *Fugu*, mouse and human MManRs. Sequence of the human PLA2R region containing CTLDs 3–5 was used as an outgroup. T_hm _– time of separation between human and mouse [96 Myr; 60], T_fish _– time of separation between ray-finned and lobe-finned fishes [430 Myr; 32]. Time of duplication (T_dupl_) was calculated using average between molecular clock calibrated with T_hm _and with T_fish_.

For all but two pairs of duplicated genes, Ks values estimated with four different methods (see Methods) were between 1.5–2.5, which indicates a complete saturation of the synonymous sites (Figure 4(A)). Ks values so high cannot provide an accurate estimation of the duplication age, but we can conclude with confidence that the CTLDcp gene duplications are at least 150 Myr old, which is the time required for complete saturation of silent sites assuming a mutation rate of 2.5 substitutions/silent site/billion years in fish [59]. If, however, Ks values presented in Figure 4(A) and the silent mutation rate are close to correct, the corresponding duplication timeframe is predicted to be 300–500 Myr.

In order to date the duplications based on molecular clock measurements, we aligned duplicated *Fugu *CTLD sequences with their vertebrate orthologues present in GenBank, and built linearized phylogenetic trees based on the alignments. As human and mouse sequences were invariably available, the divergence time between these two species [96 Myr; 60] was used to calibrate the clock, together with the divergence time between Actinopterygii and Sarcopterygii [430 Myr; 32]. Symmetrical tree topology ((H, M) (F, F1)), expected for a Actinopterygian-specific duplication, was revealed by at least one phylogeny reconstruction method we used for the following six homologue groups (data not shown): brevican, neurocan, MManR, SRCL, tetranectin and HAPLN3, with duplications dated 369, 284, 397, 377, 360 and 312 Myr, respectively. A typical tree with symmetrical topology is shown for MManR in Figure 4(B). The other six alignments (aggrecan, versican, layilin, attractin, C1qRP, CRTL1) produced trees with topologies suggesting a duplication predating the split between Actinopterygian and Sarcopterygian. The portion of symmetrical topologies (50%) in the CTLD set is similar to the ratio reported by Taylor and coworkers in fish: 15 of 27 (55 %) [61], and 25 of 53 (45%) [62] for bigger and more heterogeneous gene collections.

## Discussion

### Draft assembly limitations

A systematic study based on draft-quality whole-genome data for an organism like *Fugu rubripes *has some limitations, as the genomic sequence is incomplete, fragmented and sometimes misassembled, and the expressed sequence information is scarce. On the other hand, many of the genomes that are currently being sequenced will be released and remain for sometime in the same state as the *Fugu *genome data are now. Indeed, more than a year after the initial release [31] very few improvements to the *Fugu *genomic data [v.3 assembly and EST sequencing project; [63]] have been published. Therefore, it is essential to extract useful biological information from draft-quality whole-genome sequences. Our study is such an attempt.

We have mentioned four limitations of the draft-state assembly – incompleteness, fragmentation, misassembly and lack of expression information. While the last might appear the biggest problem, we found that *ab initio *predictions combined with manual curation and interspecies comparison have proven to be very accurate (e.g. see PKD1L example), thanks to the compactness of the *Fugu *genome, smaller ratio between intron and intergenic region sizes compared with mammalian genes, wealth of data for comparative analysis etc. We do not expect that sequencing the remaining 5% of the *Fugu *genome, which is mostly heterochromatic regions, will lead to discovery of many new CTLDcps. From the comparison of the *Fugu *CTLDcp repertoire discovered by us and found in other fish species independently, the only surprising omission in our results is a MBP orthologue. MBP sequences have been found in several other fish species. Their absence in Fugu may represent a bona fide gene loss. As to the fragmentation, only a few of the CTLDcps are split between scaffolds, namely versican and some MManR paralogues (Figure 1). All of the fragmented genes are big, and in most cases the fragments can be combined easily to reveal the full sequence. Finally, misassembly signs were observed in several CTLDcp loci while comparing two versions of the assembly. These showed as presence of repeated regions in the v.2 assembly, which disappeared in the v.3 assembly.

### Two groups identified in higher vertebrates are not detectable in *Fugu*

We could not detect CTLDcp representatives for groups V (NK cell receptors) and VII (lithostathine) in the *Fugu *genome. CTLDs in the members of these groups have lost their carbohydrate-binding activities, and perform functions that have, apparently, evolved after evolutionary separation of tetrapods from fish, or which are mediated by other proteins in fish. For example, group VII members are secreted into the digestive tract – a system that is very flexible evolutionally. Group V is probably one the youngest and most rapidly evolving sets of CTLDcps; its component members vary significantly even between rodents and human, a phenomenon connected to the co-evolution with the acquired immune system proteins that group V CTLDcps interact with.

Our conclusion on the absence of group V CTLDcps in the *Fugu *genome is at odds with the conclusions of two studies describing group V CTLDcp evolution in chordates. A recent paper describes possible CD94 homologues (cichlid killer cell lectin receptor, cKLR) in bony fishes *Paralabidochromis chilotes *and *Oreochromis niloticus*, which are encoded by a large multi-gene family with at least 10 members [28]. Another recent work described sequencing of a CD94 homologue in a tunicate [64].

**Figure 5:**
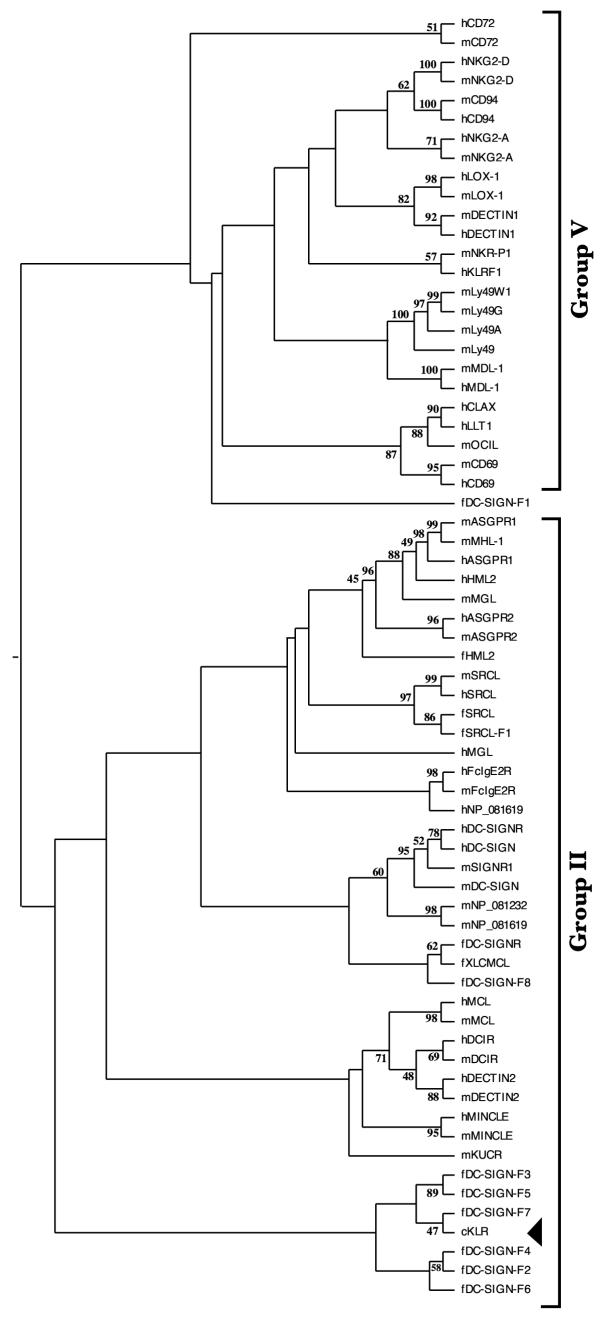
**Relationships between fish, mouse and human group V and II CTLDs. **Non-redundant set of CTLD sequences from known human and mouse CTLDcps classified as groups II and V, *Fugu *CTLDcps classified as group II, and putative killer cell receptor from *Paralabidochromis chilotes *(cKLR) were aligned with ClustalW. A consensus phylogenetic tree was built from 100 bootstrap trials using the *protdist *(with PAM distance matrix) and *neighbor *programs from the PHYLIP package. Black triangle shows position of cKLR. Bootstrap values higher than 40 are indicated.

The decision by Sato et al. [28] to assign putative fish killer cell receptors to group V rather than to group II was based on several considerations, including gene structure, absence of canonical Ca^2+^/carbohydrate-binding residues, and phylogenetic analysis based on the CTLD alignment. The latter consideration is mentioned as the most important one. However, as the authors themselves note, bootstrap values for placing cKLR on the group V branch, are "low to moderate". Indeed, we found that in phylogenetic trees built using different methods (maximum parsimony, distance estimation method with PAM matrix followed by neighbor-joining tree reconstruction, maximum likelihood) from the ClustalW alignments of cKLR sequences with group V and group II CTLD sequences from *Fugu*, mouse and human, cKLR placement is unstable. As shown in Figure 5, on a tree built by the neighbor-joining method we found cKLR on the branch containing the *Fugu*-specific subset of group II CTLDcps (DC-SIGN-F1 – DC-SIGN-F8), most of which do contain residues required for Ca^2+^/carbohydrate binding. On a tree built by the maximum parsimony method, we found cKLR on a separate branch equally related to group II and group V sequences (not shown). Also, a BLAST search with the complete cKLR sequence (GI 31789959) in the non-redundant NCBI protein database returns members of the ASGR subgroup of group II as top matches. Therefore, we judge that sufficient support for assignment of cKLR to group V is lacking and the question of the presence of the NK-cell receptor family in fishes is still open.

As to the putative CD94 homologue from tunicates, it is indeed more similar to CD94 than to any other CTLDcp. However, the low level of sequence homology and the lack of evidence for existence of group V CTLDcps in more advanced taxa does not allow a confident statement that the sequence from tunicates is a CD94 orthologue, rather than a result of convergent evolution.

### Expansion of the innate immunity CTLDcp groups in *Fugu*

Unlike pairwise unlinked duplications (see below), tandem duplications and other gene family expansions are limited to two groups, namely the DC-SIGN subgroup of group II and MManR. In mammals, members of these subgroups play an important role in innate immune responses. In particular, DC-SIGN is actively studied due to its ability to bind and internalize a broad range of bacterial and viral pathogens, including HIV-1 and *Mycobacterium tuberculosis *(reviewed in [65]), while MManR is also implicated in binding and phagocytosis of a wide range of microorganisms [66]. Expansion of these groups, most notably the DC-SIGN subgroup, in *Fugu *may reflect a larger role for innate immunity in host defense in lower vertebrates. Interestingly, multi-copy clusters comprising at least 10 genes encoding close cKLR homologues were identified in another cichlid fish species *Oreochromis niloticus *[28], which suggests another parallel between the expanded DC-SIGN subgroup in puffer fish and cKLRs of cichlids.

There are no extra members, however, in the *Fugu *collectin group – another CTLD group directly involved in innate immunity in mammals. Moreover, the mannose binding protein (MBP), which is the best-studied mammalian collectin involved in lectin complement activation pathway, was not detected by us. The absence of MBP orthologues in *Fugu *is rather puzzling, as MBP sequences have been found in several other fish species (*Danio rerio*, *Cyprinus carpio *and *Carassus auratus*; [20]), and are well conserved within the *Cyprinidae *carp family. The collectin family is also present and expanded in the Urochordate *Ciona intestinalis *with nine collectin genes identified in the draft genome sequence [67], although it is not clear whether one of these nine genes is an MBP orthologue. Given the role of MBPs in complement activation in mammals, and their presence and level of conservation in the carp family, it is possible that the *Fugu *MBP orthologue does exist but is not covered by the draft genome sequence. Complement-activating C-type lectins from lower organisms have been identified but not completely sequenced [68]; they have multiple CTLDs as in CPL-III from the protochordate *Clavelina picta *[69] or lack the collagen domain and show more similarity to other CTLDcps such as the glucose-binding lectin (GBL) from another tunicate, *Halocynthia roretzi *[70].

### *Fugu *dual CTLD molecules – a missing link between vertebrate and invertebrate CTLDs?

Previous whole-genome studies of the CTLD superfamily in two invertebrates [13,14] failed to identify any groups of CTLDcps common to both invertebrates and vertebrates. A group of predicted dual CTLD-containing proteins in *Fugu *(F1) may be the first vertebrate group that has detectable homologues in invertebrates. Alternatively, it is possible that none of the *Fugu *F1 group members are in fact orthologous to the invertebrate sequences, as sequence similarities are only moderate (~30% ID) and the domain architecture is simple and could have evolved independently in different lineages. However, several observations suggest that at least F1 members from *Fugu *and zebrafish and SCARF proteins from *Girardia tigrina *evolved from the same predecessor. First, similarity levels between fish sequences and between fish and planarian sequences are about the same. It is unlikely that the fish sequences are unrelated, which implies that F1 members are evolving quickly, and only major structural features of these molecules are under selective pressure [49]. Second, the CTLDs of planarian and, in all cases at least one CTLD of the fish dual-CTLDcps, contain residues characteristic of Ca/carbohydrate binding. In vertebrates, ability to bind carbohydrates is associated with the oldest CTLDcps groups, and is considered to be an ancestral feature of the CTLD. Indeed, both vertebrate CTLDcp groups that we failed to find in *Fugu *(V and VII) have lost sugar-binding properties. This is also the case for the antifreeze proteins from fish and snake venom CTLDcps, which have only been found in the corresponding clades. Third, similar domain organization (two CTLDs, no transmembrane domain) is also observed in two other known groups of invertebrate CTLDcps: immulectins from various insect species [71,72] and nine proteins from *C. elegans*, classified as group D1 by Drickamer and Dodd [13]. Despite identical domain structure, none of these proteins shows statistically significant homology to the fish F1 group members or their putative homologues from planarian or *Drosophila*. Altogether, this indicates that domain structure alone cannot establish an evolutionary link between the fish and invertebrate sequences. Hence, the suggestive link between the F1 group fish members and the planarian and *Drosophila* proteins is even more interesting.

### CTLDcp classification update

The existing classification of CTLDcps is generally accepted and popularly used in studies of the superfamily and recently has been updated [15]. The classification divides CTLDcps into monophyletic groups of proteins with identical overall domain architecture based on a combination of structural and phylogenetic information. Although two previous large-scale studies [13,14] showed it to be inapplicable for description of invertebrate CTLDcps, our analysis of the puffer fish genome indicates that it is sufficient to describe the superfamily in all vertebrates, with only minor modifications and some extensions.

Our newly discovered CTLDcps, with a few exceptions, do not fit into the existing classification because of their unique domain architecture. We propose several new groups to accommodate the novel CTLDcps which have been found in both higher and lower vertebrates and are supported by cDNA sequences:

• XV – Bimlec (type I transmembrane protein), which in phylogenetic trees is not placed on the same branch as group VIII sequences, has a distinct exon-intron structure of the CTLD region and a neck not similar to the neck region of the group VIII sequences;

• XVI – SEEC, based on unique domain architecture;

• XVII – CBCP, based on unique domain architecture;

Additional groups may be required for the sequences not supported by sufficient expression data (NLSLH) and other sequences from the "unclassified" group whose presence in higher vertebrates is not clear. Also, clade-specific groups, such as fish antifreeze proteins (AFP), dual-CTLD sequences (group F1) predicted by us and so far identified only in fish, or snake venom CTLDcps which lack orthologues in other vertebrates, are required.

It has been suggested previously [19,48] that AFPs belong to group VII based on their domain architecture and exon-intron structure. However, our phylogenetic analysis of an alignment of CTLD sequences from all known human and mouse CTLDcps and 26 different fish CTLD-containing protein sequences identified by searching the NCBI protein database with BLAST, indicates that they constitute a phylogenetically distinct group including all known soluble fish CTLD-containing proteins, except Cyprinidae collectins. As to the exon-intron structure, introns in the group XII (EMBP) CTLDs are at exactly the same positions as in group VII and AFP-like CTLDs, which suggests that all three groups are closely related but does not allow classification of the fish AFP-like sequences to either of the mammalian groups. Interestingly, just like most of the AFPs, mammalian EMBPs contain an atypical WIGL motif with a glycine in the fourth position, a substitution not observed in any other mammalian CTLD we analyzed. Taken together, these observations indicate that in a broader evolutionary perspective the differences between some of the groups including CTLDcps with a very similar domain architecture (VII, XII and AFP; II and V) become less distinct, which makes classification of the "intermediate" or "ancestral" sequences, equally related to more than one group, problematic.

### Selective duplication of the *Fugu *CTLDcp-encoding genes and the whole-genome duplication hypothesis

The hypothesis that whole-genome duplications were one of the main driving forces in vertebrate evolution, providing genetic material for increased diversity and progressive development [73], and that there were two rounds of whole-genome duplication in vertebrate phylogeny (the 2R hypothesis) [73,74], is actively debated [75,76]. A more recent whole-genome duplication is suggested for the Actinopterygian branch [61]. Ray-finned fish are the most diverse group of vertebrates, and based on the initial observation that each of the four human HOX gene clusters has two homologues in zebrafish [77] it was suggested that they have undergone an additional round of a whole-genome duplication after the divergence from Sarcopterygian about 430 Myr ago [61]. Analysis of the genome duplication in fish can give a picture of a duplicated genome after 300–400 Myr of evolution and fill the gap between the now generally accepted recent tetraploidizations in plants [78] and yeast [79] and the alleged more ancient duplication(s) of the ancestral vertebrate genome.

Although many fish genes are indeed duplicated [61,77,80-82], it is not clear whether the copies were created by a complete genome duplication (autopolyploidy), merge of different genomes (allopolyploidy), regional duplication, or simply a series of tandem duplications. Attempts to show that ancient tetraploidization (has not) occurred usually involve: (i) searching for an excess of paralogue groups where the number of members is double the number of alleged duplications (i.e. 2 in case of Actinopterygian duplication, and 4 in case of vertebrate duplication, the "one to four rule") [74,76]; (ii) showing that a statistically significant number of duplications took place at approximately the same time by molecular clock estimation or synonymous substitution counting [83,84]; (iii) using phylogenetic methods to assess the relation between duplication and speciation events [61]; and (iv) showing that duplicated genes are arranged in paralogous blocks on chromosomes (paralogons) [62,85,86]. We used these approaches to analyze the nature of the observed CTLDcp duplications in *Fugu*.

Our results clearly show that tandem gene copying is a mechanism of CTLD family evolution and led to generation of three gene clusters: DC-SIGN-F2 – DC-SIGN-F5 (4 genes), CRTL1-F1 and CRTL1-F2, and AFPL-F1 and AFPL-F2. Members of the two latter clusters are nearly identical and may be an assembly artifact. Twelve other duplicated genes are not linked in the current assembly and have sequences much more diverged than tandem duplicates. Of the 12 genes only MManR, which has 3 paralogues, is present in more than two copies. We consider this is important evidence in favor of a whole-genome duplication, as sporadic duplications cannot explain such a strong bias towards two-member paralogue groups. Unfortunately, the results of duplication time estimations are less conclusive as they give only a broad timeframe for the possible duplication events of about 300–400 Myr. As in the case of some other fish gene families reported previously [61,62,87,88], molecular phylogeny reconstruction performed by us often indicates that duplications occurred before the divergence between fish and tetrapods. However, this could be an artifact of the method caused by different selection pressures on duplicates. Unfortunately, there is practically no overlap between vertebrate and invertebrate CTLD families, so we could not use invertebrate sequences to refine phylogenetic analysis. To conclude: phylogenetic relationships between CTLD paralogues and estimated duplication time distribution indicate that there was a burst in duplication activity in the *Fugu *genome 300–400 Myr ago. While we cannot determine definitively the nature of the duplications (tandem, regional or whole-genome), a pronounced bias in the number of two-member paralogue groups strongly suggests that there was a single large-scale or whole-genome duplication event in fish.

Another interesting observation is that CTLDcp genes were either duplicated, or retained after a large-scale duplication, in a pronounced selective manner. One group (I) is duplicated completely, while in other groups only partial duplications are found. Interestingly, group I (lecticans), which in tetrapods contain four large (>2000 amino acids) proteins, very similar to each other in sequence and domain structure, is a good candidate for demonstrating the 2 R hypothesis. If the four lecticans arose as a result of the alleged two rounds of the whole-genome duplication early in vertebrate history, the fact that the family was also completely duplicated in fish and retained after the duplication appears very non-random and implies some functional explanation. In the human genome, all four genes encoding lecticans are located on different chromosomes (1, 5, 15 and 19), but it is not clear whether they are linked in *Fugu*.

Another group that conforms to the 2 R hypothesis is group VI, which in tetrapods has four members with almost identical domain structure in mammals (Pla2R, MManR, DEC-205 and Endo180). Though in the *Fugu *genome we identified 7 group VI sequences, some of which are fragmented (Figure 1), phylogenetic analysis shows that only one member of the family (MManR) was quadruplicated, while others are present in a single copy. Both molecular clock and Ks-based methods date the MManR duplications at approximately the same time as other CTLDcp gene duplications. Phylogenetic trees, built on alignment of the overlapping portions (Figure 1) of the complete sequences and three largest fragments (fMManR-F1, fMManR-F2, fMManR-F3) have symmetrical structure, with fMManR-F1, fMManR-F2 and fMManR-F3 forming a separate branch (Figure 4(B)). A whole-genome duplication, generating fMManR and fMManR-F1, followed by tandem duplications of fMManR-F1, producing fMManR-F2 and fMManR-F3, can explain this topology.

## Conclusions

We have performed an analysis of the CTLD superfamily composition in *Fugu rubripes*. Although the sequence assembly is in the draft state and lacks physical mapping information and native cDNA sequences that could be used to make and verify gene predictions, the quality of the data is good enough despite these limitations to answer many important questions. Our study demonstrates that all but two groups of CTLDcps present in mammals are also found in fish, that most of the groups have the same composition as in mammals, and that the missing groups are the evolutionarily most dynamic ones involved in physiological processes that may be specific to higher vertebrates. We also identified at least one distinct fish-specific CTLD group, which could be the first known vertebrate CTLD group also found in invertebrates.

The compactness of the *Fugu *genome makes it an extremely convenient reference sequence for identification of new genes based on supporting similarity features, and we were able to identify and predict the structure of several new CTLD-containing genes highly conserved between *Fugu *and human. The new sequences are supported by cDNA and EST sequences from databases and have previously unknown domain architectures. We are now characterizing some of these sequences experimentally. We also show that CTLDcp-encoding genes are selectively duplicated in *Fugu*, in a manner that suggests an ancient large-scale duplication event in fish.

## Methods

Corrected gene predictions are made available as a distributed annotation system (DAS) [89] resource [90], which can be viewed in the EnsEMBL genome browser. The data source names for predictions based on assemblies v.2 and v.3 are fugu_ctld_1 and fugu_ctld_2, respectively. Transcript sequences (in FASTA format) for the CTLDcp-encoding genes created or modified by us (stable IDs starting with ANU) and their translations are also provided in the additional file 1 and additional file 2, respectively.

Searches and gene annotations were done on version 2 of the *Fugu rubripes *genome assembly [31] downloaded from the EnsEMBL web site [91,92]. When the third version of the assembly was released, we mapped gene annotations onto it. Mapping was done on the basis of SSAHA [93] matches in the v.3 assembly for exons predicted on the v.2 assembly. The v.2 assembly is currently accessible at the Singapore IMCB site [94] and on our server [95], which is pre-configured to display the DAS track with our annotations and contains a reference table with hyperlinks for all of the *Fugu *CTLDcp genes discussed. Version 3 of the assembly can be found on the main EnsEMBL web site [34]. The EnsEMBL genome browser can be easily configured to display our gene models as a DAS track.

We used a multi-step approach to find genes encoding CTLDs. First, a hidden Markov model (HMM) profile of the CTLD was used to scan a FASTA database of EnsEMBL-predicted genes with the hmmsearch program from the HMMER package [96]. To detect orthologues and paralogues, the set of *Fugu *sequences found was compared with the 95% non-redundant set of sequences of human CTLDcps that could be found in the Entrez proteins database, using the Inparanoid program [40]. All of the 25 orthology links detected by Inparanoid were checked manually.

Because of systematic and sporadic errors in EnsEMBL gene predictions, we had to manually revise the structure of each of the 69 genes encoding proteins detected by the HMM-based search. This was done using the Apollo genome annotation software [97] connected to a local installation of the EnsEMBL database. To facilitate annotation, several additional feature tracks were added to the EnsEMBL database:

a) Similarity features detected by GeneWise [98] search of *Fugu *scaffold sequences with a CTLD HMM built in a global alignment mode. This was done to detect well conserved CTLDs while avoiding many false positives.

b) Same as a), but with an HMM built in the local alignment mode; this was done to detect highly conserved fragmented CTLDs;

c) Similarity features detected by a TBLASTN search of *Fugu *scaffold sequences using all known human CTLD sequences; this was done to detect CTLDs that are less conserved;

d) ORFs encoding putative transmembrane (TM) domains. To create this track a database of all possible ORFs longer than 45 bp was produced and translated into protein sequence using the EMBOSS programs. This was then scanned with the TMHMM program [99] to detect ORFs that encode putative TM domains.

To verify whether there are CTLDs that were not covered by EnsEMBL gene predictions, we searched for all significant CTLD similarity features detected by GeneWise which do not overlap with any of the genes analyzed in the first stage. This step led to detection of 25 new CTLD-coding genes, including most of the ones that have previously uncharacterized domain organization. At the next stage we analyzed the loci with different CTLD similarity features detected by genewisedb search with a local alignment HMM. Finally, the features identified by BLAST and not overlapping with already detected genes were analyzed. This set of features mostly contained only partial CTLD matches.

We translated both the new and already predicted gene CDSs into protein sequences and performed another Inparanoid comparison. Phylogenetic relationships were analyzed with the programs from the Phylip package [100]. ClustalW [101] guiding trees were used for quick phylogeny estimation and in cases where a proper multiple alignment could not be made.

BioPerl [102] and EnsEMBL Perl modules were used to automate all stages of the analysis. Domain architectures were analyzed with the SMART web service [103].

To estimate the proportion of substitutions in synonymous sites, we aligned translated sequences of the duplicated CTLDcp-encoding genes with ClustalW, using either whole sequence or sequence for the CTLD-encoding region only, and built nucleotide sequence alignments based on the protein alignments. Ks estimations were performed with four methods: Lynch and Connery [104] and Li [105], both implemented in the ntdiffs package [104]; and Nei and Gojobori [106] and Yang and Nielsen [107], both implemented in the yn00 program from the PAML package.

Duplication dating using the calibrated molecular clock approach was performed as in [83]. Alignments of CTLD-containing regions of *Fugu *paralogues and their mammalian orthologues were made with ClustalW. The MEGA2 program [108] was used to build linearized trees from Poisson-corrected distances, p-distances and Gamma-corrected distances by the neighbor-joining method with 1000 bootstrap samplings. The global clock was calibrated using divergence times 96 Myr and 430 Myr for human-mouse and fish-mammal splits, respectively [32,60,83].

## List of abbreviations

AFP, antifreeze protein;

AFPL, AFP-like;

CBCP, Calx-β and CTLD-containing Protein;

CDS, coding sequence;

CETM, CTLD, EGF, TransMembrane domain

CTLD; C-type-lectin-like domain;

CTLDcp; CTLD-containing protein;

DAS, distributed annotation system;

EMBP, eosinophil major basic protein;

EST, expressed sequence tag;

HMM, hidden Markov model;

MBP, mannose-binding protein;

Myr, million years;

NLSLH, Novel L-SeLectin Homologue;

PKD1, polycystic kidney disease protein 1;

SEEC, SCP, EGF, EGF, CTLD;

TM, transmembrane.

## Authors' contributions

ANZ carried out the bioinformatics studies and participated in the interpretation of its results. JEG conceived the study and participated in the interpretation of its results. Both authors participated in writing the manuscript and approved its final form.

## Supplementary Material

Additional File 1 **Transcript sequences for re-annotated *Fugu *CTLD genes. **The file contains cDNA sequences (in FastA format) for all CTLDcp-encoding genes that were re-annotated by us (sequence identifiers starting with ANU). Click here for file

Additional File 2 **Protein sequences for re-annotated *Fugu *CTLD genes. **The file contains protein product sequences (in FastA format) for all CTLDcp-encoding genes that were re-annotated by us (sequence identifiers starting with ANU). Click here for file
